# Harness the power of the DOI: Digital object identifiers and what they can do for you

**DOI:** 10.1002/cl2.1063

**Published:** 2019-12-02

**Authors:** Sarah Young

Evidence from Campbell reviews and similar research is valuable because of its potential impact on policy‐making and real‐world practice. Practitioners and policy‐makers search the internet every day for information to help them make decisions. Therefore, raising the online visibility of this research evidence is critical.

As researchers, there are concrete, and relatively simple steps we can take to both improve the reach of our work and to help us measure impact. Leveraging the “digital object identifier” (DOI) can more effectively connect evidence consumers to evidence producers.

DOIs are a widely used and well‐established standard for directing readers to digital information objects in a permanent, consistent way. Briefly, DOIs are governed by the International DOI Foundation, made up of registration agencies like Crossref and DataCite, which facilitate the assignment of DOIs to information objects of an academic and scholarly nature like journal articles, datasets, books and book chapters. These permanent digital identifiers were established due to the dynamic and ephemeral nature of the digital landscape, in which information can quickly be lost as URLs change and objects move around the internet. If and when the URL of a journal article or other work changes, the DOI infrastructure allows for that change to be accounted for, behind the scenes, such that users will consistently be directed to the object when they click on a DOI, even if that object has moved to a new website.

Case in point: recently, Campbell moved all of its scholarly content, including systematic reviews and protocols, to the Wiley Online Library platform. While the URLs are now different, the DOIs associated with those articles have not changed. Rather, the metadata underlying the DOI has been updated to reflect the new location of the article. Thus, any link to a Campbell review via the DOI will be correctly directed to the full text content at Wiley.

This DOI infrastructure creates a network of scholarly information that promotes openness of scholarly citation data, facilitates attribution and provides the means to more effectively share and disseminate scholarly research output. Collaborations and partnerships in this scholarly ecosystem strengthen the growing network by linking distributed products of academic research, authors and institutions through other persistent scholarly identifiers, such as ORCID.

As a researcher, you can leverage the power of DOIs to promote your work and more effectively connect readers to the full text content of your publications and other products of research, like datasets. When referring to your work in social media like Twitter or in blog posts, and when citing your own work and the work of others, be sure to include the DOI. For Campbell articles, the DOI is built into the URL, so simply sharing the link in the citation is enough. As the internet inevitably changes over time, your work remains discoverable and the links between your research and the broader body of knowledge remain intact.

DOIs also facilitate the measurement of impact through attention metrics like numbers of views and downloads. Tools like Altmetric and PlumX rely on DOIs to capture data related to content usage across platforms like Twitter, ResearchGate, Mendeley, Wikipedia, blogs, and publisher websites. Wiley provides a link from each Campbell systematic review and protocol to an Altmetric profile for that work, lending insights into readership and usage, including uptake in policy documents, news and other academic sources. Mentions in social media can provide you an immediate sense of the impact of your work, where traditional citations can take months, if not years, to emerge in the scholarly literature.

The Wiley platform provides a number of tools to facilitate the use of DOIs in citations and social media. For example, from an article page in the Campbell Systematic Review journal, you can click on Tools to access an Export Citation feature, which will include a DOI as part of a properly formatted citation. The Share feature on article pages will allow you to quickly post to Twitter, LinkedIn and other social media, in a way that builds in the DOI for easy tracking by alternative metrics tools.

As the scholarly information ecosystem continues to expand exponentially, and information becomes increasingly fluid and dynamic across the digital landscape, getting reliable research evidence into the hands of decision‐makers is all the more important. DOIs, when used effectively, can help you as a researcher sustain the links between your work, related research and highly visible and utilized information resources like Wikipedia. By simply including the DOI in any and all references to your work, be it a tweet, a blog post, or another journal article, you can help improve the visibility and usage of high quality research evidence.

